# Supporting structural biologists in Africa requires resources and capacity building

**DOI:** 10.1038/s41594-024-01438-9

**Published:** 2024-12-01

**Authors:** Emmanuel Nji, Aurélien F. A. Moumbock, Katharina C. Cramer, Nicolas V. Rüffin, Jamaine Davis, Oluwatoyin Asojo, Julia J. Griese, Amma A. Larbi, Michel N. Fodje

**Affiliations:** 1 BioStruct-Africa, Alfred Nobels Allé 37C, LGH 1001, 14152 Huddinge, Sweden; 2 BioStruct-Africa, 10 Link Road, Kwame Nkrumah University of Science and Technology (KNUST), Kumasi, Ghana; 3 Department of Parasitology and Microbiology, Centre for Research in Infectious Diseases, P.O. Box 13591, Yaoundé, Cameroon; 4 Institute of Pharmaceutical Sciences, Albert-Ludwigs-Universität Freiburg, Freiburg, Germany; 5 University of Bonn, Center for Advanced Security, Strategic and Integration Studies, Römerstr. 164, 53117 Bonn, Germany; 6 Department of Biochemistry, Cancer Biology, Neuroscience & Pharmacology, Meharry Medical College, 1005 Dr. DB Todd Jr Blvd, Nashville, TN 37208; 7 Dartmouth Cancer Center, One Medical Center Drive, Lebanon, NH 03756; 8 Department of Cell and Molecular Biology, Uppsala University, SE-751 24 Uppsala, Sweden; 9 Department of Biochemistry and Biotechnology, College of Science, Kwame Nkrumah University of Science and Technology, PMB, Kumasi, Ghana; 10 Canadian Light Source, Inc, University of Saskatchewan, 44 Innovation Boulevard, Saskatoon SK S7N 2V3, Canada

Structural biology beats at the heart of modern science. It reveals the molecular mechanisms underlying disease processes, facilitating drug and vaccine development, and improving existing therapies. Beyond healthcare, it has an important role in agriculture, biotechnology, food safety and environmental sustainability. Therefore, structural biology is integral to achieving the United Nations Sustainable Development Goals (SDGs), including Good Health and Well-being (SDG 3), Zero Hunger (SDG 2), and Clean Water and Sanitation (SDG 6). The recent award of the 2024 Nobel Prize in Chemistry jointly to Demis Hassabis and John Jumper of Google DeepMind for their pioneering work on protein structure prediction and the development of AlphaFold, and to David Baker for his groundbreaking contributions to protein design, reveal how central structural biology is to scientific progress.

Structural biology is particularly important in Africa, since the continent faces significant health challenges, including neglected diseases, a high burden of infectious diseases, droughts and lack of clean water. By understanding the mechanisms of action of drug targets such as malaria parasite sugar transporter protein, researchers can create more effective therapies^1,2^. Similarly, structural studies of a *Mycobacterium tuberculosis* enzyme have paved the way for new treatments for drug-resistant strains. Structures of the malaria parasite's chloroquine resistance transporter protein have helped researchers understand how the parasite develops resistance to chloroquine. This breakthrough will enable development of methods to restore chloroquine's effectiveness in treating malaria^3^. Structural biology aids in vaccine design, as seen with COVID-19^4^. Outside healthcare, structural biology techniques have helped address agricultural issues, like disease-resistant crops^5^, environmental sustainability^6^ and plastics degradation for environmental remediation^10^.

However, the development of structural biology *in and for* Africa is hampered by significant barriers. These include non-existent or limited access to necessary scientific infrastructure, challenges in educating and training scientists and technicians, restricted career prospects, a limited pool of skilled personnel, and a persistent brain drain of qualified experts. In recent decades, structural biology has advanced through automation, enabling many experiments to be conducted remotely at specialized facilities, though these are often located in the Global North (e.g., Africa remains the only continent without a synchrotron light source, with utilised synchrotron light sources such as Diamond Light Source in the United Kingdom). AI-assisted tools like AlphaFold have opened even more new possibilities, dramatically reducing reliance on costly, location-specific facilities, democratizing access, and somewhat leveling the playing field for scientists in previously marginalized regions. However, as groundbreaking as AlphaFold is, there is a catch: the technology is only useful if scientists know how to use it. The growth of structural biology in Africa requires more than just access to tools—it demands investment in people, training, and basic infrastructure. This is where capacity building becomes essential.

BioStruct-Africa (www.biostructafrica.org) is a grassroots initiative aimed at bridging this gap. Founded in 2017 with the goal of empowering African scientists, BioStruct-Africa is actively engaged in capacity building, focusing on training researchers in structural biology techniques, including AI-driven tools like AlphaFold. The initiative follows a bottom-up approach, fostering the next generation of African structural biologists through workshops, mentoring programs, and collaborative networks. BioStruct-Africa’s efforts are already yielding promising results on the ground. A workshop titled *Hands-on Training in Structural Biology: A Tool for Sustainable Development in Africa* (Series 5) was recently held in Douala, Cameroon (7-11 October, 2024). The workshop focused on utilizing AlphaFold for protein structure prediction and applying AlphaFold2-generated structures to screen small molecule compound libraries for drug discovery. The demand for training was overwhelming, with five times more applicants than available seats, meaning that many qualified and motivated applicants had to be turned away due to limited resources, highlighting a pressing need for resources to maximize the reach of this program.

When asked about their interest in the workshop, their current work, and how they plan to apply the knowledge gained, the answers of the applicants offer valuable insights into the dynamics within an emerging community of structural biologists on the African continent. On the one hand, the majority expressed that participation would expand their knowledge, allow them to acquire new skills, and enhance their professional portfolios. Many also emphasized their intention to apply these skills in practical contexts, such as drug discovery and therapy development. On the other hand, participants linked their desire for career advancement with a broader, community-centered narrative, indicating a strong willingness to both locally implement this new knowledge should resources become available and to thwart the persistent brain drain. While the community shared a common motivation for joining the workshop, their research areas are thematically diverse from agriculture and food safety to antibiotic resistance and neglected diseases. Some areas that applicants are interested in include Lassa virus, East Coast fever and the use of medicinal plants for alternative drug development. This wide range of specializations reflects both the broad expertise of applicants and Africa's unique and urgent health challenges.

The applicants in this workshop represented a diverse range of academic levels, from master’s students to senior lecturers, and spanned thirteen different nationalities, painting a vibrant picture of an emergent scientific community of African structural biologists. Recent scholarship has identified such dynamics as crucial for developing strong scientific capacities^7^. First, the successful implementation of large-scale scientific research infrastructures, such as the African Light Source (AfLS) in the future, will require robust scientific networks and ecosystems. These infrastructures serve as collaboration hubs, enabling researchers to access cutting-edge technology and exchange knowledge, thus advancing scientific discovery. Without a well-established network of skilled researchers, these infrastructures may not reach their full potential^8^. Second, emerging scientific networks provide the essential foundation for significant advances in science, with well-connected scientific communities fostering the exchange of ideas, mentorship, and interdisciplinary collaboration— all key drivers of innovation^9^.

Africa has the potential to lead in structural biology, by applying it to solve its own scientific challenges and contribute to global breakthroughs, given the right support and adequate resources. Sustained investment is key. So far, capacity-building in structural biology across the African continent is heavily dependent on public funding, much of which is still provided by major European and U.S.-based funding organizations. These funds are typically awarded to individuals, emerging research communities, networks, or institutions, to establish their own laboratories and hire, train, and develop scientists and technicians. However, the high demand highlights a substantial funding gap that needs to be taken seriously by key stakeholders, including African governments and research funding bodies.

Capacity building efforts of BioStruct-Africa’s^10,11^,which started in 2017, have highlighted a widespread lack of awareness about the field’s techniques and potential in many institutes and universities. This knowledge gap is a bottleneck that needs to be addressed. Basic infrastructure is lacking – from essential software for interpreting and visualizing protein structures to sufficient hard disk memory on researchers’ personal computers. Having a synchrotron light source in Africa is a long-term goal that has the potential to transform scientific research on the continent. The future of structural biology in Africa is bright, but realizing its full potential will require concerted effort. Until we have a synchrotron in an African country, a combination of cutting-edge technology, such as AlphaFold or synchrotron remote access, and grassroots capacity-building initiatives like BioStruct-Africa can serve as bridges to enable Africa to participate in—and contribute to—global scientific advancements.

The adage "Give a man a fish, and you feed him for a day; teach a man to fish, and you feed him for a lifetime." is well worth keeping in mind. Investing in capacity building is the next and crucial step towards sustainable advancement in this vital field on the African continent.
